# A United Nations General Assembly Special Session for Mental, Neurological, and Substance Use Disorders: *The Time Has Come*

**DOI:** 10.1371/journal.pmed.1001159

**Published:** 2012-01-17

**Authors:** Judith K. Bass, Thomas H. Bornemann, Matthew Burkey, Sonia Chehil, Lenis Chen, John R. M. Copeland, William W. Eaton, Vijay Ganju, Erin Hayward, Rebecca S. Hock, Rubeena Kidwai, Kavitha Kolappa, Patrick T. Lee, Harry Minas, Flora Or, Giuseppe J. Raviola, Benedetto Saraceno, Vikram Patel

**Affiliations:** 1 Department of Mental Health, Johns Hopkins Bloomberg School of Public Health, Baltimore, Maryland, United States of America; 2 Mental Health Program, The Carter Center, Atlanta, Georgia, United States of America; 3 Department of Psychiatry and Behavioral Sciences, Johns Hopkins Hospital, Baltimore, Maryland, United States of America; 4 Division of Child and Adolescent Psychiatry, Dalhousie University, Halifax, Canada; 5 Department of International Health, Johns Hopkins Bloomberg School of Public Health, Baltimore, Maryland, United States of America; 6 Division of Psychiatry, University of Liverpool, Liverpool, United Kingdom; 7 World Federation for Mental Health, Woodbridge, Virginia, United States of America; 8 Johns Hopkins School of Medicine, Baltimore, Maryland, United States of America; 9 Department of Medicine, Massachusetts General Hospital, Boston, Massachusetts, United States of America; 10 Centre for International Mental Health, Melbourne School of Population Health, University of Melbourne, Victoria, Australia; 11 Program in Global Mental Health and Social Change, Department of Global Health and Social Medicine, Harvard Medical School, Boston, Massachusetts, United States of America; 12 Department of Psychiatry, University of Geneva, Geneva, Switzerland; 13 Global Initiative on Psychiatry, Hilversum, The Netherlands; 14 Faculty of Epidemiology and Population Health, London School of Hygiene and Tropical Medicine, London, United Kingdom; 15 Sangath, Goa, India

## Abstract

Vikram Patel and other global mental health leaders call for a special session of the UN General Assembly to discuss and debate action needed on mental, neurological, and substance use disorders, which have been left off the international NCDs agenda.

**Summary Points**

- Mental, neurological, and substance use (MNS) disorders are leading causes of the global burden of disease and profoundly impact the social and economic well-being of individuals and communities.
- The majority of people affected by MNS disorders globally do not have access to evidence-based interventions and many experience discrimination and abuses of their human rights.
- A United Nations General Assembly Special Session (UNGASS) is needed to focus global attention on MNS disorders as a core development issue requiring commitments to improve access to care, promote human rights, and strengthen the evidence on effective prevention and treatment.

## The Burden of MNS Disorders

Mental, neurological, and substance use disorders and related disabilities (MNS disorders) are leading contributors to the burden of disease globally. (The term MNS is a relatively new acronym coined by the World Health Organization [WHO] to refer to the complete range of disorders of the brain and the mind for its mental health Gap Action Programme [mhGAP].) Unipolar depressive disorders, alcohol use disorders, and self-inflicted injuries rank within the top 20 leading causes of burden of disease (as measured by disability adjusted life years [DALYs]) across all age groups and neuropsychiatric disorders, collectively, account for 22% of DALYs for women aged 15–59 years [1]. Even in the least developed regions of the world where infectious and parasitic diseases are prominent, MNS disorders are a major source of DALYs lost [1]. Over the next 20 years, it is estimated that neuropsychiatric disorders alone will account for the loss of an additional US$16.1 trillion with “dramatic impacts on productivity and quality of life” [2]. Suicide claims the lives of at least 1 million people annually [3] with over 85% of suicides occurring in low- and middle-income countries (LMICs). Nearly 4% of all deaths globally are attributable to alcohol [4]. While many MNS disorders begin in childhood and adolescence (when an individual is being educated, establishing effective social relationships, and preparing for work), the burden associated with dementia and stroke is expected to accelerate with increasing life expectancies around the world. Currently, it is estimated that about 25 million people have dementia; this number is expected too reach over 80 million by 2040, with nearly three-quarters of affected people living in LMICs [5].

MNS disorders are associated with low rates of treatment, poor treatment adherence, and increases in risky behaviors, thus influencing the risk of and outcomes in other noncommunicable (NCD) and communicable diseases. This association is bi-directional. For example, depressive disorders markedly increase the risk for NCDs such as diabetes, coronary artery disease, stroke, and dementia, and comorbidity of depression and NCDs complicates treatment and worsens prognosis for both [6]. Stigma and discrimination are frequently experienced by people with MNS disorders, showing similarities to the experience of those with stigmatizing diseases like HIV. Indeed, people with MNS disorders suffer some of the worst abuses of human rights in modern times [7]. Conflict, displacement, poverty, gender-based violence, and other social determinants of ill health increase the risk for MNS disorders [8],[9], and MNS disorders are, in turn, associated with worsening of social and economic circumstances, setting up a vicious cycle of poverty and illness.

## Addressing the Burden of MNS Disorders

We have robust evidence on cost-effective interventions that can be adapted to meet the needs of people affected by a range of MNS disorders in different contexts and cultures [10],[11], and we have confirmation that effective delivery of such evidence-based interventions is possible even in contexts with limited resources [12]–[14]. We have evidence that early detection and treatment can be affordable and would have considerable benefits for people, families, and communities. For example, more than 65% of people with epilepsy can be successfully treated with affordable drugs; in most countries the cost of treatment can be as low as US$5 per patient per year [15]. Research has shown that, in addition to improving health states, the treatment of MNS disorders also improves economic well-being of the affected person [16], and mounting evidence underscores the importance of treatment of MNS disorders for community- and state-level economic and political development [17]. Nevertheless, an enormous treatment gap persists throughout the world, particularly in the least developed countries.

A number of challenges need to be addressed in a global strategy for reducing the burden of MNS disorders. These challenges include inadequate resource allocation, cultural and contextual influences on the manifestations of illness [18], stigma and discrimination, shifting and imprecise diagnostic systems, limited etiological evidence, limited effectiveness of interventions for the prevention and care of some conditions, and the continued sequestration of mental health in siloed parellel systems apart from general health services. While there is a need for both specialty care as well as for decentralization of services to substantially expand access to treatment and care, in many countries the treatment and care for MNS disorders is predominantly in specialist hospitals in urban centers [19], far from where most people live, resulting in care for only a small fraction of people with MNS disorders.

The time has come for recognition at the highest levels of global development, namely the UN General Assembly, of the urgent need for a global strategy to address the global burden of MNS disorders.

## The Goals of the Proposed UNGASS

The proposed UNGASS aims to position MNS disorders as a global development priority. The declaration presented at the UNGASS should call for governments, multilateral agencies, and donors to mobilize resources to implement actions to address the burden of MNS disorders. Three broad areas of action need urgent investment.

The first is to enhance access to evidence-based packages of care, for example, by widely implementing the WHO mhGAP guidelines [20] for the treatment of MNS disorders. The delivery of these interventions will need task-shifting approaches and integration of mental health care within primary health care systems and in other sectors, such as education and social welfare. We will need to strengthen health systems and ensure equitable access for marginalized populations such as persons displaced by conflict and those who are socially disadvantaged.

Second, the human rights commitment enshrined in the Convention on the Rights of Persons with Disabilities [21] should be realized to ensure that people with MNS disorders live a life with dignity. There should be no place in our world for placing people with MNS disorders in chains, or locking them away from their communities. Prisons should never be the de facto care settings for people with MNS disorders. The capacity of people with MNS disorders to advocate for themselves must be strengthened and supported.

Third, actions must be taken to expand our knowledge about MNS disorders. Even as advances in genetics and neuroscience are improving our understanding of MNS disorders, there have been no major innovations in the prevention or treatment for any MNS disorder in the past two decades. A specific fund is needed, perhaps modeled along the lines of the Drugs for Neglected Diseases Initiative, to fast-track discovery of new pharmacological, psychological, and social treatments and preventive interventions. The fact that MNS disorders affect people in all countries should offer considerable incentive for investments by both public and private sectors in this initiative.

Securing the commitment of a majority of governments for a UNGASS will require a concerted effort from the diverse group of stakeholders concerned with MNS disorders. Above all, persons affected by MNS disorders and their families must be empowered to play a leading role in advocating for their well-being and rights. The Movement for Global Mental Health (http://www.globalmentalhealth.org/) and World Federation for Mental Health (http://www.wfmh.org/) will work together with the WHO and other multilateral and civil society agencies to rally support. Recently, the Board of the World Federation for Mental Health (WFMH) formally adopted a position to prioritize the “grand challenge” for global mental health related to transforming health system and policy responses [22]. Building on its recent efforts to advocate for mental health in the global health agenda on NCDs—such as the presentation of position statements at the World Health Assembly meeting and the WHO meeting for health ministers in Moscow—WFMH has launched its “Great Push for Mental Health” program. This program includes the development of a “People's Charter for Mental Health” that intends to translate stakeholder identified priority needs into practical actionable steps for country implementation. This charter will be developed in consultation with the organizations from 96 countries who have signed up to the “Great Push” initiative so far, representing over one million people including consumers, family members, advocates, researchers, professional organizations, and policy makers. Together, this grand coalition of local, national, and global actors will converge their energies towards the implementation of a UNGASS to achieve the ultimate goal of reducing the global burden of MNS disorders.

## Abbreviations

MNS: mental, neurological, and substance use
NCD: noncommunicable disease
UNGASS: United Nations General Assembly Special Session
WHO: World Health Organization
